# Etiology and effusion characteristics in 29 cats and 60 dogs with pyothorax (2010‐2020)

**DOI:** 10.1111/jvim.16699

**Published:** 2023-04-26

**Authors:** Lynelle R. Johnson, Steven E. Epstein, Krystle L. Reagan

**Affiliations:** ^1^ Department of Medicine and Epidemiology University of California‐Davis Davis California USA; ^2^ Department of Surgical and Radiological Sciences University of California‐Davis Davis California USA

**Keywords:** culture, cytology, empyema, multidrug resistance

## Abstract

**Background:**

Pyothorax, an accumulation of inflammatory fluid in the pleural space, is often caused by foreign body inhalation in dogs, whereas the etiology in cats can be more difficult to discern.

**Objective:**

Compare clinical, microbiologic findings, and etiology in cats and dogs with pyothorax.

**Animals:**

Twenty‐nine cats and 60 dogs.

**Methods:**

Medical records of cats and dogs diagnosed with pyothorax from 2010 to 2020 were reviewed. Clinical findings, fluid analysis, and microbiologic results were retrieved.

**Results:**

Antimicrobials had been administered to equal proportions of cats and dogs before fluid sampling (45% and 47%). Groups did not differ in age or total protein concentration or percentage neutrophils in pleural fluid, but effusion cell count was significantly higher in cats than in dogs (*P* = .01). Neutrophils containing intracellular bacteria were identified in more cats (27/29, 93%) than dogs (44/60, 73%; *P* = .05). Penetrating damage to the thorax was implicated as the cause of pyothorax in equal percentages of cats (76%) and dogs (75%). Etiology could not be determined in 2 cats and 1 dog. Cats had higher numbers of bacterial isolates per patient (median, 3) than dogs (median, 1; *P* = .01) and anaerobes were isolated more often in cats (23/29, 73%) than in dogs (27/60, 45%; *P* = .003).

**Conclusions and Clinical Importance:**

Pyothorax had similar etiologies in cats and dogs. Cats had higher fluid cell counts, higher numbers of bacterial isolates identified per patient, and intracellular bacteria detected more commonly than did dogs.

**Abbreviations**
FIP: feline infectious peritonitis
MDR: multidrug resistance
TMS: trimethoprim sulfamethoxazole

## 1 INTRODUCTION

Pyothorax in cats and dogs is characterized by intrapleural accumulation of proteinaceous fluid with high white blood cell count, large percentage of inflammatory cells, and variable types of bacteria. Most infections in dogs and cats are polymicrobial,^1^, ^2^ but some studies have found single organism infection.^3^, ^4^ Diagnosis of bacterial pyothorax is often readily achieved because of the presence of intracellular bacteria on cytology in 68% of dogs and 91% of cats.^2^ However, empirical use of antimicrobial agents before sample collection could impact the diagnostic utility of both cytology and culture.

The underlying cause of pyothorax varies based on geography, environment, and the species involved. Hunting dogs in rural environments are most commonly affected by grass awn migration,^5^, ^6^, ^7^ which causes pyothorax as well as pneumothorax and often requires surgical intervention.^8^ Cats do not inhale foreign material commonly,^7^, ^9^ and various studies have indicated poor identification of etiology in cats.^10^, ^11^ Bite wounds and other penetrating injuries as a cause for pyothorax are supported by the findings that outdoor access and multi‐cat households are common in affected cats,^10^, ^11^ but parapneumonic spread also has been suggested as a frequent cause of pyothorax.^12^

Retrospective studies are limited in ability to determine the etiology of disease. Hence, our primary aim was to comprehensively evaluate historical findings and combined cytological and microbiological features of pyothorax in cats compared to dogs and assess the ability to confidently determine a cause for empyema in the 2 species. We hypothesized that penetrating damage to the thorax (e.g., bite wounds, foreign bodies, traumatic injuries) would be the most common causes in both dogs and cats and that evidence for underlying pleuropneumonia would be uncommon. Our secondary aim was to summarize antimicrobial susceptibility data for infecting bacteria.

## 2 MATERIALS AND METHODS

Ours was a retrospective study that collated data from review of medical records. Using the search terms pyothorax, empyema, and pleural infection, the medical record database at the William R. Pritchard Veterinary Medical Teaching Hospital at the University of California‐Davis was searched between 2010 and 2020 for cats and dogs with a clinical diagnosis of pyothorax. Pyothorax was defined as a suppurative exudative pleural effusion with protein concentration ≥2.5 g/dL or cell count ≥5000/μL.^13^ Thoracic fluid was described as septic when bacteria were identified on cytologic assessment by a veterinary clinical pathologist, regardless of microbial culture findings. Inclusion criteria for the study included contemporaneous collection of pleural fluid for cytology and microbiologic culture (aerobic and anaerobic). Animals with specimens collected at disparate times, those with surgically collected swabs, or those with culture results from only tissue specimens were excluded from analysis. Animals with aseptic chylothorax or neoplastic effusions identified on cytology by a board‐certified clinical pathologist were not included in the study. Cats considered likely to have effusion related to feline infectious peritonitis (FIP) based on very high fluid protein concentration (above the exudative range) with cell counts ≤5000/μL or positive immunohistochemistry on pleural fluid also were excluded.

Fluid specimens were obtained by thoracocentesis or from an indwelling thoracic catheter that had been placed using aseptic technique. Thoracic fluid was placed in both EDTA and nonanticoagulant tubes for transport to the laboratory, and any sample obtained after midnight was stored at 4°C for <8 hours before processing.

Fluid analysis was performed by staff of the clinical pathology laboratory and cytologic assessment was completed by a board‐certified pathologist. Total nucleated cell count was performed using an automated cell counter (Advia 120, Siemens, Deerfield, IL) and a 100‐cell differential cell count was performed. Slides were prepared using an automated cell stainer (Model 7151 Wescor Aerospray Hematology Pro, ELITech Bio‐Medical Systems, Logan, UT) and Wright‐Giemsa stain. One milliliter of fluid was transferred to a disposable tube and centrifuged for 5 minutes at 2400 rpm. A drop of the supernatant was applied to the refractometer for assessment of total protein concentration. Total protein concentration in pleural fluid (g/dL), total cell count (cells/μL), and differential cytology (%) were recorded for all cases and cytologic reports were reviewed for intracellular bacteria (neutrophils containing phagocytosed bacteria), which was taken as evidence of septic pleural effusion.

Fluid specimens were submitted for aerobic and anaerobic bacterial cultures in all cases, and selected cases were submitted for isolation of *Mycoplasma* species at the discretion of the attending clinician. One drop of pleural fluid was plated onto 5% defibrinated sheep blood and MacConkey agars (Hardy Diagnostics, Santa Maria, CA) and incubated in the presence of 5% CO_2_ at 35°C for isolation of aerobic organisms. Pre‐reduced anaerobic *Brucella* blood agar (Anaerobe Systems, Morgan Hill, CA) was used for anaerobic culture with incubation at 35°C under anaerobic conditions. Pleuropneumonia‐like organism base with thallium acetate and penicillin G (University of California‐Davis Media Laboratory, Davis, CA) was used for *Mycoplasma* species isolation with incubation at 35°C in 5% CO_2_. Standard biochemical methods were used to identify cultured bacteria. The number and species of isolates obtained from each cat and dog were recorded under aerobic and anaerobic conditions. Cases were designated as polymicrobial culture if ≥2 species of bacteria were isolated.

Bacterial susceptibility testing was performed according to standards established by the Clinical Laboratory Standards Institute using broth microdilution.^14^ Susceptibility interpretations of minimum inhibitory concentration (MIC) testing were based on animal standards when available and, when not available, interpretative criteria established for human medicine were used. Multidrug resistance (MDR) was defined as resistance to at least 1 antimicrobial in ≥3 classes of antimicrobial drugs in which the wild type bacteria could be susceptible to (eg, no intrinsic resistance).

Interviews with owners, physical examination findings, thoracic imaging studies, and results of surgical, bronchoscopic, or histologic investigations were reviewed. Patient age and information about administration of antimicrobials within 1 week before presentation were collected from the medical record. In cats, FeLV/FIV status was determined from the medical record. All thoracic imaging studies (e.g., radiography, ultrasonography, computed tomography) were interpreted by a board‐certified radiologist, and results were used by the clinician of record in case management. Imaging was retrospectively evaluated by 1 of the authors (Lynelle R. Johnson) as part of record review in determining etiology and to assess the side of the effusion. Contemporaneous notes made by the primary clinician along with the assessment of results were used to assign the probable cause of pyothorax, which was defined as penetrating damage to the thorax (e.g., foreign body inhalation, bite wound, traumatic injury), iatrogenic pyothorax, suspected pneumonic spread of infection, or systemic disease‐ associated pyothorax according to criteria presented in Table 1. Animals were placed into specific categories when ≥2 or were met within that category and other causes could be rationally excluded.

**TABLE 1:** Probable etiology of pleural effusion in cats and dogs was determined by interrogation of owner history, physical examination findings, and diagnostic imaging as well as interventional procedures.

*Bite wound* ^a^
Known history of fighting
Multi‐animal household
Previous abscess
Thoracic wound
*Foreign body*
Identified surgically or bronchoscopically
Pyogranulomatous inflammation with foreign material or migrating tracts
*Traumatic injury* ^b^
Focal radiographic infiltrate
Outdoor/exposure to foxtails
Isolation of *Actinomyces*
Thoracic wound
Coincident pneumothorax
*Pulmonary disease* ^c^
Previous history of pneumonia
Risk factors for aspiration
Parenchymal infiltrates on radiographs
Histopathologic evidence of parenchymal inflammation (pneumonia)
*Systemic disease*
No identifiable local cause of empyema in an animal with systemic inflammatory response, an infected surgical implant, or bacteremia
*Iatrogenic*
Esophageal feeding tube placement errors
Postsurgical infection
Post‐thoracocentesis infection
*Unknown*

^a^ Animals with 2 or more criteria were included in this etiologic group.

^b^ Animals that did not have surgery performed but that had 2 or more features compatible with a penetrating injury were included in this etiologic group.

^c^ Animals with 2 or more criteria with no known history of fighting were included in this etiologic group.

Cases were assigned the etiology of foreign body inhalation when foreign material was identified by surgery, bronchoscopy, computed tomography or magnetic resonance imaging, or when histopathology indicated birefringent material, inflammatory tracts, or focal pyogranulomatous inflammation. Bite injuries were documented by evaluation of the clinical history and physical examination findings. In cats, a known history of fighting, outdoor access, living in a multi‐cat household, history of cat bite abscess or thoracic wound in the past 6 months contributed to the probable diagnosis of a bite wound as the cause of pyothorax. Animals with pyothorax were assigned the likely cause of traumatic injury (outside‐in or inside‐out) when no surgical intervention had been performed or histopathology was available and there was no history of an abscess but at least 2 of the following criteria were met: the animal had outdoor access, there was known exposure to plant awns, a thoracic wound was present, focal radiographic infiltrates resolved with treatment, coincident pneumothorax was identified, or *Actinomyces* was isolated from pleural fluid. Animals in this category also had no historical, clinical, or imaging evidence of pneumonia, systemic disease, or iatrogenic causes.

Pneumonia or pulmonary disease as an underlying cause for pyothorax was assigned to animals lacking airway foreign bodies that had radiographic or computed tomographic evidence of parenchymal infiltrates with air bronchograms and without volume loss, or those with histologic evidence of parenchymal inflammation or pneumonia. Aspiration pneumonia was included in this group and was considered a probable diagnosis if previously identified risk factors including vomiting, recent anesthesia, laryngeal disease or enteral nutrition were reported.^15^, ^16^

Systemic disease was considered the underlying cause of pyothorax in animals with multiorgan infectious disease or systemic inflammatory response syndrome, the loss of localized control of disease resulting in a circulating inflammatory response likely mediated by cytokines.^17^ Evidence of immunosuppression as a contributor to disease was examined in these cases by assessing coincident administration of immunosuppressive medications. Iatrogenic causes were identified in animals with procedural interventions potentially involving the pleural space such as thoracocentesis, thoracotomy, or esophageal feeding tube placement that preceded the onset of pyothorax. Finally, the etiology was considered unknown if a reasonable clinical explanation for pyothorax could not be obtained from information available in the medical record.

Statistical analysis: Clinical data were assessed for normality using the D'Agostino and Pearson test and are presented as mean ± SD for normally distributed data or median and range for nonparametric data. Age, duration of illness, circulating white blood cell count, pleural fluid cell counts, pleural fluid protein concentration, and fluid percentage of neutrophils were compared between cats and dogs using Student's *t*‐test for normally distributed data or Mann‐Whitney *U* test for nonparametric data. Isolation of anaerobes, presence of sepsis in pleural fluid, and presampling exposure to antimicrobials were compared between cats and dogs using Fisher's exact test. Significance was set at *P* < .05. (GraphPad Prism v 9.5.1, San Diego, CA).

## 3 RESULTS

**FIGURE 1:**
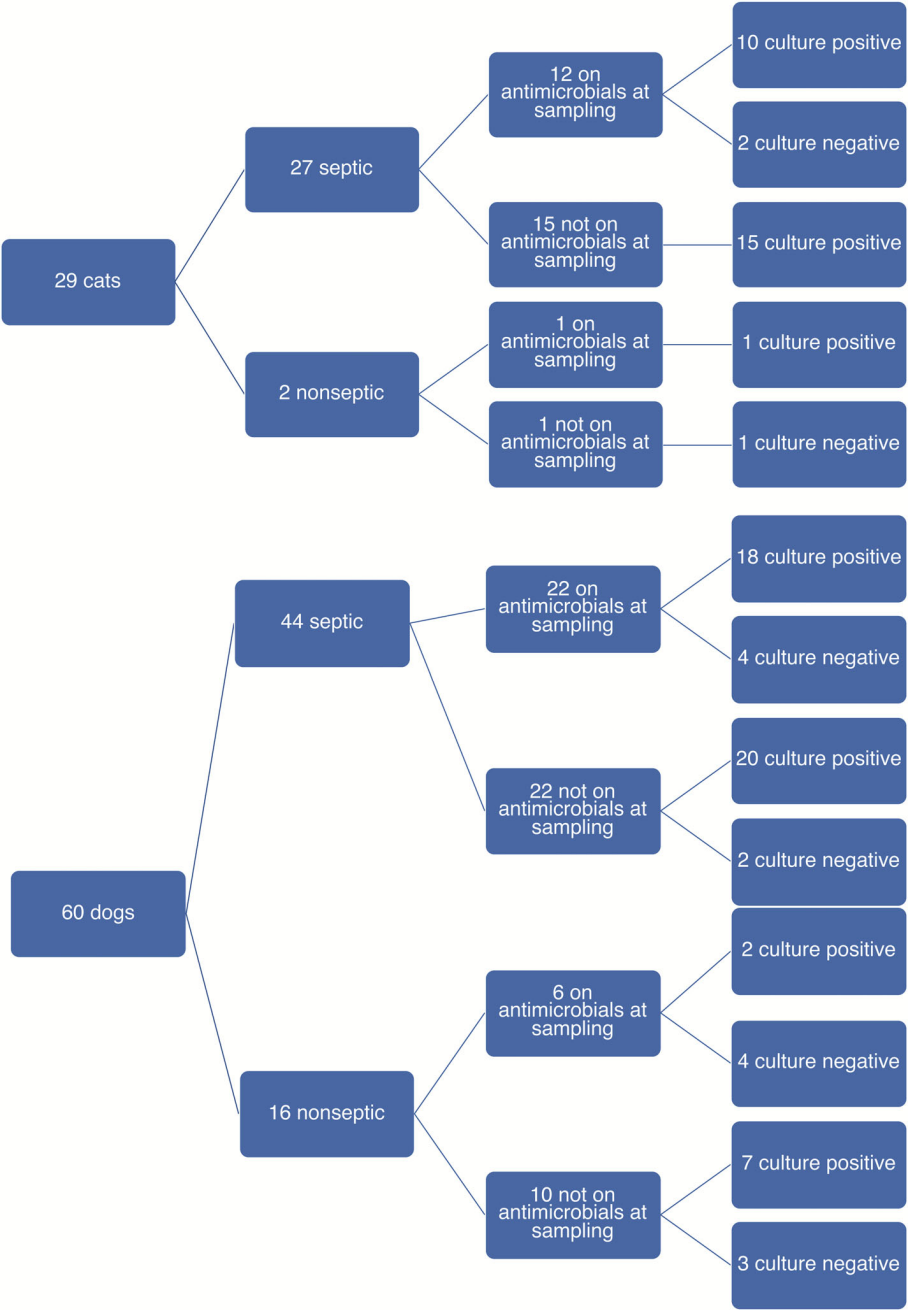
A total of 29 cats and 60 dogs had pleural fluid analysis and microbial cultures submitted. Septic pleural fluid was detected more commonly in cats than in dogs although antimicrobials had been administered before fluid sampling in equal proportion of cats and dogs.

Search of the medical record data base yielded 60 cats and 100 dogs with a clinical diagnosis of pyothorax. Pleural fluid culture had not been performed in 30 cats and 40 dogs, and 1 cat was diagnosed with FIP, yielding a total of 29 cats and 60 dogs for further evaluation. Antimicrobials had been administered before fluid sampling in equal proportions of cats (13/29, 45%) and dogs (28/60, 47%; *P* = 1.0; Figure 1). In cats, a penicillin‐type drug or enrofloxacin was given most frequently and the combination was provided to 7 cats. Cefovocin had been administered to 3 cats and metronidazole to 1 cat (in combination with a penicillin and fluoroquinolone). In dogs, penicillin‐type drugs (n = 18) and enrofloxacin (n = 12) also were the most commonly administered antimicrobials, with 6 dogs receiving combination treatment. Cats received combination antibiotic treatment before pleural fluid sampling significantly more often than did dogs (*P* = .02). Four dogs were treated with doxycycline, 3 with a cephalosporin, 2 with metronidazole, and 1 with amikacin.

Groups did not differ in age (cats: median, 8.0; range, 2‐17.5; dogs: median, 5.25; range, 0.3‐13; *P* = .06; Table 2). In cats, 18 males were affected (2 intact and 16 neutered) compared to 11 female spayed. In affected dogs, 33 were female (5 intact and 28 spayed) and 27 were male (9 intact and 18 neutered). Median weight of dogs was 26.6 kg (range 1.1‐78 kg) with 7 dogs <10 kg, 11 dogs 10 to 20 kg, 28 dogs 20.1 to 35 kg, and 12 dogs 35.1 to 78 kg. Domestic cats (22/29) and mixed breed dogs (12/60) were affected most commonly. Labrador retrievers (n = 10) and German Shepherds (n = 6) were over‐represented compared to the hospital population. The FeLV/FIV status was assessed in all cats with 1 cat testing positive for FeLV. Groups did not differ in duration of illness or circulating white blood cell count between cats and dogs or across causes of disease (*P* > .05; Table 2). Thoracic radiographs available for review in 27/29 cats identified unilateral effusion in 2 cats and bilateral effusion in the remaining cats. In dogs, thoracic radiographs were reviewed for 53 cases and unilateral effusion was identified in 3 dogs.

**TABLE 2:** Clinical features in dogs and cats with pyothorax.

	Cat					Dog					
	Bite wound	Foreign body	Traumatic injury	Pulmonary	Iatrogenic	Bite wound	Foreign body	Traumatic injury	Pulmonary	Iatrogenic	Systemic
n	10^a^	3	9^b^	3	2	2	31	12	4	3	7^b^
Age years	6 (2.5‐14)	6 (4‐13.5)	8 (2‐12)	13 (2‐17.5)	11 (8‐14)	5 (1‐9)	5 (1‐12.5)	4 (1‐9)	8.25 (6‐12)	3 (0.3‐8.5)	5.5 (3‐10)
Duration of illness days	3.5 (2‐45)	7 (7‐11)	4 (2‐31)	7 (5‐20)	5 (4‐6)	6	7 (1‐30)	5 (1‐30)	3.5 (2‐35)	5 (2‐40)	6 (1‐21)
Circulating WBC/μL	18 406 (9620‐26 310)	29 650 (21 950‐39 080)	25 750 (4600‐56 110)	21 790 (15 554‐51 840)	19 337	38 005 (9030‐66 980)	18 080 (4550‐47 010)	16 455 (11 650‐45 510)	11 620 (6460‐37 990)	6475	23 370 (1100‐35 476)
Effusion protein concentration (g/dL)	5.4 (3.4‐6)	3.6 (2.9‐4.2)	4.5 (3.0‐5.9)	4.9 (3.4‐5.0)	3.25 (2.7‐3.8)	3.3 (2.3‐4.3)	4.4 (2.9‐6.3)	4. 6 (3.6‐5.2)	3.8 (1.8‐4.7)	2.8 (1.9‐3.0)	4.2 (1.8‐4.8)
Effusion WBC/μL	195 720 (53 280‐3 460 000	409 695 (53 280‐346 000)	344 000 (295 830‐523 560)	146 300 (106 000‐677 200)	19 850 (3700‐36 000)	102 560	120 600 (21 300‐530 220)	186 700 (35 420‐443 700)	24 800 (8250‐159 420)	8000 (900‐11 900)	57 500 (14 180‐141 700)
% neutrophils	95 (75‐96)	80 (1‐94)	90 (66‐99)	83 (80‐84)	86 (75‐97)	95 (95‐96)	83 (56‐97)	85 (70‐92)	84 (69‐92)	94 (77‐95)	86 (62‐95)
Septic pleural fluid	9/10	3/3	9/9	3/3	1/2	1/2	25/31	9/12	2/4	2/3	4/7

*Note*: Numbers are presented as median with range. In 2 cats and 1 dog, no etiology for pyothorax determined; however, septic pleural fluid was documented.

^a^ One cat was FeLV positive.

^b^ One cat and 5 dogs were known to be receiving corticosteroids.

**FIGURE 2:**
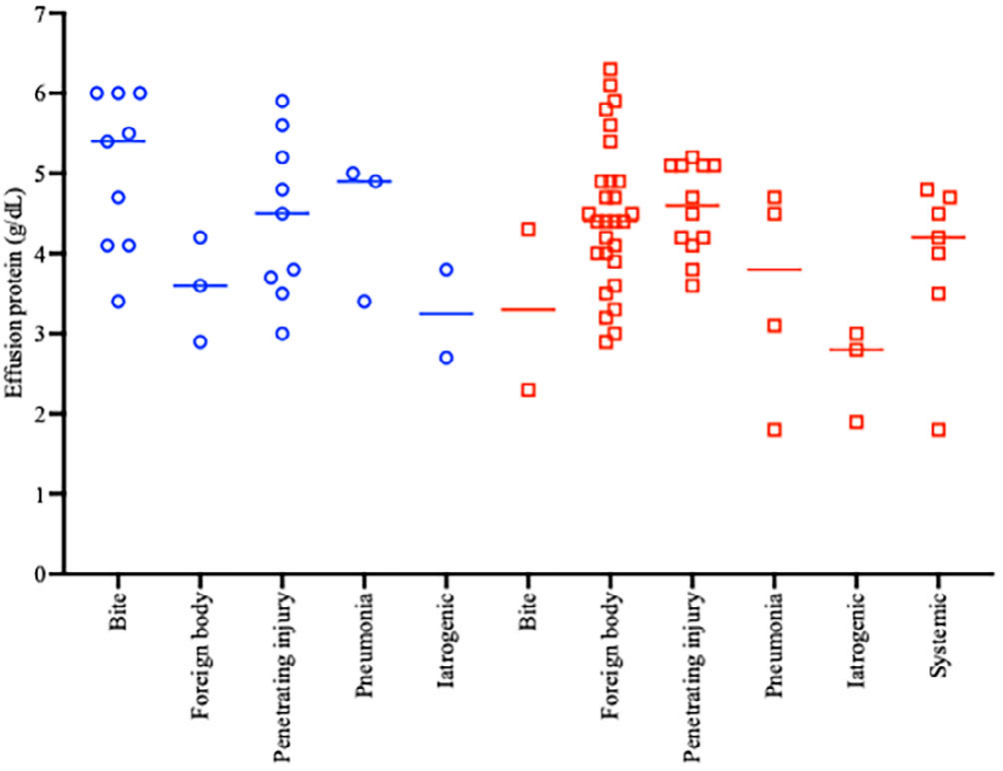
Total protein concentration in pleural effusion (g/dL) for assigned causes of pyothorax in cats (blue circles) and dogs (red squares). Horizontal bars represent median values.

**FIGURE 3:**
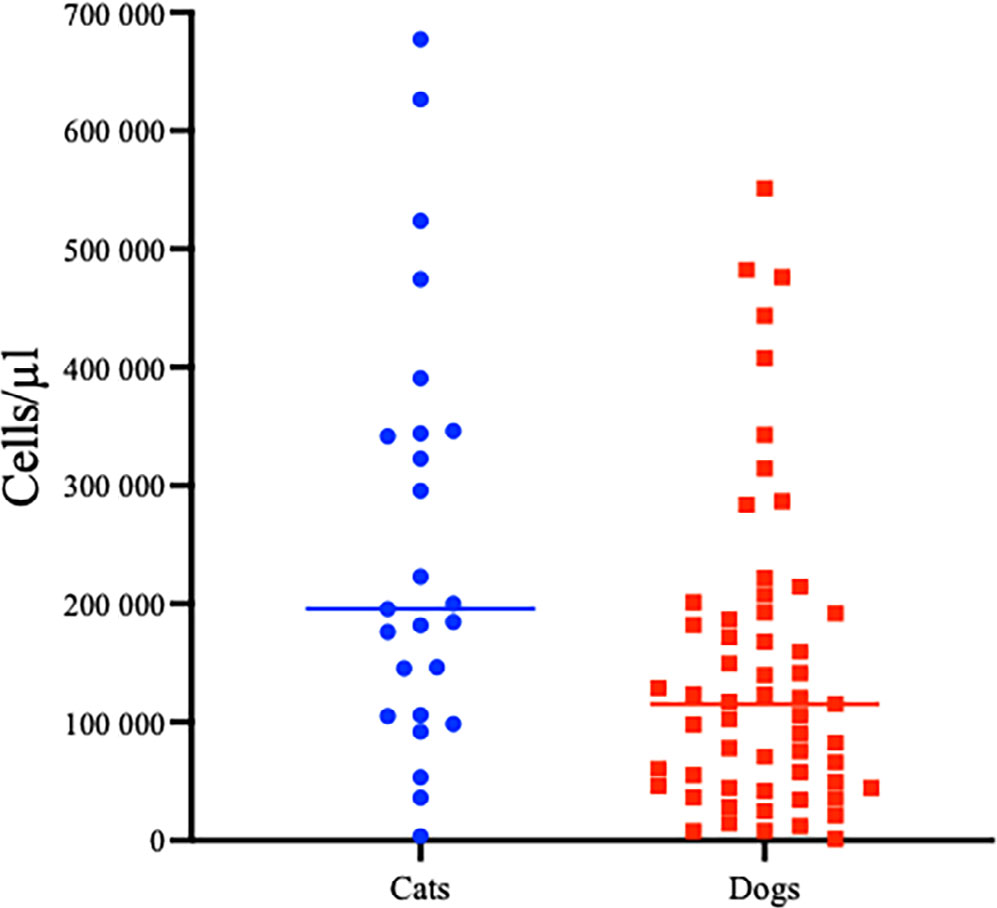
Total nucleated cell count (medians illustrated by horizontal bars) in pleural fluid from cats (blue circles) was significantly higher than in fluid from dogs (red squares) with pyothorax, *P* = .01.

Groups did not differ in total protein concentration in pleural fluid analysis (cats, 4.5 ± 1.0 g/dL; dogs, 4.3 ± 1.0 g/dL; *P* = .32). The distribution of results among different causes of pleural effusion is presented in Figure 2. Groups differed in effusion cell count (cats: median, 195 720/μL; range, 3700‐667 200/μL) vs dogs (median, 115 160/μL; range, 900‐551 520/μL; *P* = .005; Figure 3). One dog had a cell count (900/μL), below the recognized cut‐off for an exudate (5000 cells/μL)^13^ and this dog had iatrogenic pyothorax after thoracotomy. Cats and dogs did not differ in percentage neutrophils for all causes of pyothorax (median, 85% and 84% in the cats and dogs respectively; *P* = .76; Table 2).

Septic pleural fluid was detected significantly more often in cats (27/29, 93%) than in dogs (44/60, 73%; *P* = .05; Table 2). Of 13 cats receiving antibiotics at the time of sampling, 12 had septic pleural effusion and 11 had positive microbial growth whereas 22/28 dogs receiving antibiotics had septic effusion and 20 had positive microbial growth (*P* > .05; Figure 1). Aerobes were isolated from equal numbers of cats (66%) and dogs (60%), with *Pasteurella* spp. and *Actinomyces* sp. detected most commonly in cats and *Actinomyces* sp. and *Escherichia coli* most commonly in dogs (Table 3). Negative aerobic and anaerobic cultures were found in 3/29 (10%) cats and in 13/60 (22%) dogs (*P* = .10). *Mycoplasma* culture was negative in 3/3 cats and 1/1 dog tested. Cats had higher numbers of bacterial species isolated (median, 3) than did dogs (median, 1; *P* = .01), and anaerobes were isolated more often in cats (23/29, 73%) than in dogs (27/60, 45%; *P* = .003; Table 3). Polymicrobial infections were more common in cats (22/29, 76%) than in dogs (30/60, 50%; *P* = .02).

**TABLE 3:** Organisms involved in pyothorax in cats and dogs.

Organism	Cat (n = 29)	Dog (n = 60)
Aerobes	19/29 (66%)	36/60 (60%)
*Pasteurella* spp.	11	6
*Actinomyces* sp.	7	13
*Streptococcus* spp.	1	8 (biotype 3 in 5 cases)
*Staphylococcus* spp.	2	7 (*pseudintermedius* in 4, *aureus* in 1)
*Escherichia coli*		9
*Enterobacter* sp.	2	1
*Corynebacterium* sp.		2
Anaerobes	23/29 (79%)	27/60 (45%)
*Bacteroides/Prevotella* sp.	14	15
*Fusobacterium* sp.	13	16
*Peptostreptococcus* sp.	10	11
*Porphyromonas* sp.	3	4
Mycoplasma	0/3	0/1

Penetrating damage to the thorax (including bite wounds, foreign bodies, suspected penetrating injuries) was the most common probable cause of pleural effusion in both cats (22/29; 76%) and dogs (45/60; 77%; Table 2). Foreign bodies were identified surgically or bronchoscopically in 2/3 cats and 15/31 dogs. Pyogranulomatous inflammatory tracts or histologic evidence of foreign material were reported in 1/3 cats and 16/31 dogs. Six dogs had pyothorax in conjunction with pneumothorax.

Bite wounds were considered the most likely cause of pyothorax in 10 cats and 2 dogs, with external wounds evident in 2 cats and 1 dog. Fighting with neighborhood animals or among household cats was confirmed in 5/29 cats (17%) with pyothorax, 2 of which had external wounds consistent with a history of fighting at the time of diagnosis. Seven cats lived in multi‐animal (between 2 and 7 cats and dogs) households, and in 1 instance, 2 cats from the same household were diagnosed with pyothorax within a calendar year. Five cats had a history of abscess or thoracic wounds. Both dogs were from multi‐pet households and had thoracic wounds evident.

An additional 9 cats and 12 dogs were presumed to have traumatic injury to the thorax (outside‐in or inside‐out) as the cause for pyothorax. Foreign body migration could not be distinguished from bite wounds in these animals because surgical intervention was not pursued, and no histopathology was available for review. All animals had outdoor access with possible exposure to plant awns, and most were from multi‐animal household. Five cats and 3 dogs had focal radiographic infiltrates that resolved as pyothorax was treated, and concurrent pneumothorax was documented in 1 cat and 2 dogs in this group. One cat and 1 dog had thoracic wounds evident.

Three cats and 4 dogs had underlying pulmonary disease associated with pyothorax based on previous history of pneumonia (1 cat and 1 dog), risk factors for aspiration (3 cats and 1 dog), imaging findings (2 cats and 4 dogs), and histopathology (1 cat). Seven dogs had pyothorax in conjunction with systemic diseases and lacked identification of any risk factors for penetrating injury or evidence of pneumonia. Pleural fluid was septic in 4/7 dogs and cultures were positive in 5/7 dogs with 1 to 4 organisms isolated (median 1). Anaerobic bacterial cultures were positive in 4 dogs and aerobic cultures were positive in 4 dogs, with *E. coli* in 2 dogs and *Pasteurella* spp., *Staphylococcus* spp., and *Nocardia* sp. in 1 dog each. One dog had an infected orthopedic implant (*Staphylococcus aureus*) in conjunction with staphylococcal pyothorax, and no growth on culture was documented in pleural fluid from 1 case with a uterine stump pyometra. Five of these 7 dogs were receiving corticosteroids for the management of immune‐mediated hemolytic anemia (n = 3), inflammatory brain disease (n = 1), and protein‐losing enteropathy (n = 1).

Iatrogenic pyothorax was reported in 2 cats and attributed to multiple thoracocenteses in 1 cat and to a misplaced esophageal feeding tube in the second cat. Postoperative iatrogenic pyothorax was identified in 3 dogs. An etiology for pyothorax could not be determined for 2 cats and 1 dog.

In cats with bite wounds, *Pasteurella* spp. and *Bacteroides/Prevotella* sp. were the most commonly isolated organisms (n = 4) followed by *Actinomyces* sp. (n = 3) and *Porphyromonas* sp., *Peptostreptococcus* sp., or *Fusobacterium* sp. (n = 2; Table 4). Cats with bite wounds had 1 to 5 organisms isolated from pleural fluid (median, 2). *Actinomyces* sp. was isolated from 11 dogs and 1 cat with confirmed foreign bodies. In animals with probable penetrating trauma, *Actinomyces* was isolated in 3 cats and 2 dogs. *Pasteurella* spp. were isolated in 2 cats and 3 dogs, and anaerobes were isolated in 8/9 cats and 6/12 dogs. In animals with underlying pulmonary disease, aerobic culture of pleural fluid was positive for *Enterococcus* spp. and *Staphylococcus aureus* in 1 cat, *Pasteurella* sp. in 1 cat, and anaerobic bacteria in all 3 cats. In dogs with pulmonary disease, pleural fluid cultures were negative in 3/4 dogs and positive for an aerobic gram‐positive rod along with anaerobes in 1 dog.

**TABLE 4:** Aerobic and anaerobic bacteria isolated from dogs and cats with different causes of pyothorax.

		AEROBES							ANAEROBES							
		No. +	*Pasteurella* spp.	*Actinomyces* sp.	*Staphylococcus* spp.	*Streptococcus* spp.	*E. coli*	Other	No. +	*Bacteroides/Prevotella* spp.	*Fusobacterium* spp.	*Peptostreptococcus* spp.	*Porphyrmonas* spp.	Other	Total	Med
	Bite wounds															
Cat	N = 10	8	4	3	0	1	0	2	8	4	2	2	2	1	1‐5	2
Dog	N = 2	2	0	0	0	2	0	0	0	0	0	0	0	0	1	1
	Foreign body															
Cat	N = 3	2	2	1	1	0	0	0	2	1	2	0	0	0	0‐5	2.5
Dog	N = 31	20	2	11	1	5	4	1	17	8	9	6	3	5	0‐7	2
	Penetrating injury															
Cat	N = 9	7	2	3	0	0	0	0	8	3	4	4	1	1	0‐4	3
Dog	N = 12	5	3	2	0	0	0	2	6	3	2	3	0	0	0‐3	1.5
	Pulmonary disease															
Cat	N = 3	2	1	0	1	0	0	0	3	3	2	0	0	1	3‐4	3
Dog	N = 4	1	1	0	0	0	0	0	0	0	0	0	0	0	0‐4	0
	Systemic disease															
Dog	N = 7	5	1	0	1	0	2	1	2	1	0	1	0	1	0‐4	1
	Unknown															
Cat	N = 2	2	2	0	0	0	0	0	2	2	1	1	0	0	4‐4	4
Dog	N = 1	1	1	0	1	0	1	3	1	0	1	1	0	0	2	2
	Iatrogenic															
Cat	N = 2	1	1	0	0	0	0	0	0	0	0	0	0	0	0‐3	1.5
Dog	N = 3	2	0	0	3	0	1	1	0	0	0	0	0	0	1‐2	2

Susceptibility data for bacteria isolated most frequently from cats and dogs with pyothorax are listed in Table 5. All organisms showed susceptibility to vancomycin, with 93% susceptible to amikacin and 81% to gentamicin. Antimicrobials other than aminoglycosides that had the most effectiveness against aerobic organisms isolated included chloramphenicol (81%), doxycycline (79%), and trimethoprim‐sulfamethoxazole (79%). Multidrug resistant organisms were isolated from 5 dogs and 1 cat. Two dogs with bite wounds as the etiology for pyothorax had MDR *Staphylococcus pseudintermedius* and *Pseudomonas aerogenes* isolated. Neither had a history of antibiotic usage. One cat with iatrogenic pyothorax also had MDR *Pseudomonas* and *Enterobacter* species in pleural fluid with no history of previous antibiotic usage. Three dogs also had iatrogenic pyothorax, 2 of which had MDR *Staphylococcus pseudintermedius* and 1 of which had MDR *E. coli* and *Enterobacter* isolated. All of these dogs had been treated previously with antibiotics; 2 were on amoxicillin‐clavulanic acid and 1 had been treated with amikacin.

**TABLE 5:** Bacteria isolated most often from cats and dogs with pyothorax are listed with percent susceptibility of isolates to specific antimicrobials.

% susceptible	Number	Amikacin	Amoxi/Clav	Ampicillin	Cefazolin	Chloramphen	Clindamycin	Doxycycline	Enroflox	Erythromycin	Gentamicin	Imipenem	Marboflaxacin	Methicillin	TMS	Vancomycin
*Enterobacter* sp.	8	100%	75%	63%	63%	100%		88%	100%		100%	100%	100%		88%	
*Pasteurella* spp.	17	94%	100%	100%	94%	100%		100%	100%		94%	100%	100%		100%	
*Pseudomonas* spp.	3	100%							33%		100%	67%	67%			
*Staphylococcus* spp.	9	100%	22%	0%	22%	67%	22%	56%	22%	11%	56%	22%	22%	22%	44%	100%
*Streptococcus* spp.	3	33%	100%	100%	100%	67%		100%	50%	100%	33%	100%	67%		100%	100%
**All bacteria**	**40**	**93%**	**71%**	**62%**	**64%**	**81%**	**19%**	**79%**	**73%**	**19%**	**83%**	**81%**	**74%**	**22%**	**79%**	**100%**

*Note*: Shaded boxes reflect lack of interpretable data.

## 4 DISCUSSION

In our study, dogs and cats were of equivalent age and had a similar short (<1 week) duration of recognized clinical signs before presentation. Equal percentages of dogs and cats were treated with antibiotics before sampling of pleural fluid, and cats were more often treated with a combination of antibiotics. Despite this, intracellular bacteria were detected in pleural fluid more commonly in cats than in dogs, emphasizing the importance of contemporaneous cytologic assessment of exudative effusions while fluid cultures are pending. Despite equal circulating white blood cell counts in both species, cats had higher total cell counts in pleural fluid, suggesting more inflammation. Cats also had higher numbers of bacterial species isolated per patient in polymicrobial infections and a higher number of anaerobic species than dogs. Importantly, careful evaluation of the medical records identified an underlying etiology in all but 3/89 animals.

We confirmed our primary hypothesis that penetrating injury to the thorax would be the most common cause of pyothorax in both dogs and cats. Inhalation of grass awns was the most common cause for pyothorax in dogs, and likely was the reason that twice as many dogs were affected by pyothorax as cats, because cats rarely inhale foreign material.^9^ Depending on environmental conditions and geographic location, airway foreign bodies can be a common^6^, ^7^ or uncommon^18^ cause of pyothorax in dogs. Despite foreign material not being obvious in some surgical cases examined here, the presence of inflammatory tracts, as well as coincident pneumothorax, pyogranulomatous pleuritis,^19^ and isolation of *Actinomyces* sp.^20^ added to confidence in foreign body migration as the etiology in some cases.

Bite wounds were more commonly identified as an etiologic diagnosis in cats compared to dogs in our study. Although outdoor status was common in dogs and many were from multi‐animal households in both species, dogs lacked a common history of fighting or bite wounds in comparison to cats. Most cases of pyothorax in cats in our study were from multi‐cat households (61%), which has been reported to result in a 3.8‐fold increase in the risk of pyothorax, compared with cats from single‐cat households.^10^ In our study, pyothorax was diagnosed in 2 housemates that routinely fought. In a previous study of pyothorax,^10^ recent history of a bite or other external wound could be documented in only 11/76 cats (14.5%) that had a complete medical history recorded, and findings could be confirmed by external wounds in only 2 cats. However, lack of external wounds is not surprising because bites from dogs failed to leave a detectable wound in 29% of cats that had been bitten in the thorax.^21^ In another study of pyothorax, 75% of cats came from multi‐cat households, but those cats did not have a history of fighting or bite wounds and the authors could only determine an etiology for pyothorax in 3/55 cases.^11^ Most cats with pyothorax in our study had access to the outdoors (59%), similar to findings in large studies of pyothorax from Europe.^3^, ^11^ Several studies have failed to discover an underlying cause for pyothorax in cats,^1^, ^11^ whereas only 2/29 cats did not have a cause of pyothorax identified in our study.

Using the methodology described in our study, essentially equal percentages of cats and dogs had pyothorax ascribed to suspected thoracic trauma, defined as inside‐out (e.g., unrecognized foreign body, esophageal penetration) or outside‐in (e.g., thoracic trauma, bite wound) lesions as a probable cause of pyothorax. These etiologies were considered probable rather than definitive given the lack of histopathologic assessment in these cases. Isolation of anaerobes in conjunction with aerobes was common in cats with a penetrating injury as cause of pyothorax. These bacteria also are isolated from cats with pneumonia,^16^ raising the possibility of unrecognized pneumonia as a cause of pyothorax rather than suspected thoracic trauma. Isolation of *Actinomyces* sp. is often taken as evidence of foreign body‐related disease^20^ although *Bacteroides*, *Fusobacterium*, and *Actinomyces* spp. are part of the oral flora of the mouth,^22^, ^23^, ^24^ along with aerobic bacteria. Thus, these findings could indicate a bite wound as the cause of a penetrating injury. *Neisseria* sp., another oral bacterium in cats and dogs, has been isolated from bite wounds in humans,^25^ and was found in 1 cat in our study.

Detection of oral flora in cases of pyothorax also has been taken as evidence that pyothorax in cats is related to oropharyngeal aspiration resulting in pleuropneumonia.^1^, ^4^, ^12^ Although aspiration pneumonia is increasingly recognized in cats,^15^, ^16^, ^26^ cats have a well‐developed laryngospastic reflex making it less likely for infected secretions to pass from the upper to lower respiratory tract. Also, most reports of pneumonia in cats do not describe concurrent pleural involvement,^16^, ^27^, ^28^, ^29^ unless pulmonary abscessation occurs.^11^, ^30^ Therefore, although abscessation can occur with any form of pneumonia, a focal, subpleural pulmonary abscess resulting in pyothorax could reflect either a bite wound or foreign body migration rather than pneumonia.

Pleuropneumonia is relatively common in people and in horses,^31^, ^32^, ^33^ but we found low percentages of cats and dogs (5%‐10%) that could be assigned pulmonary disease as an underlying cause of pyothorax. Chronic naso‐ocular discharge was reported in 29% of cats in 1 study,^4^ and some evidence suggests that secondary bacterial infection plays a role in chronic upper respiratory tract disease.^34^ However, direct extension of nasal infection to the bronchi, lungs, and pleura would need to occur for that disease process to result in pyothorax. Given how common chronic upper respiratory tract disease without pyothorax is in cats, this etiologic mechanism would seem unlikely.

We identified 7 dogs in which pyothorax was found in conjunction with systemic disease. Microbial cultures of pleural fluid were negative in 2 dogs and monomicrobial infection was detected in 3 dogs, with multiple aerobic and anaerobic species in the remaining 2 dogs. *Staphylococcus* septicemia related to an orthopedic implant was the likely source of infection in 1 dog and use of immunosuppressive drugs may have contributed in 5/7 dogs evaluated here, but development of empyema is unusual in our experience managing septic patients. Also, large scale studies on immune‐mediated disease in dogs managed with corticosteroids failed to report pyothorax as a complication of treatment.^35^, ^36^ One dog had protein‐losing enteropathy and might have developed empyema in association with bacterial translocation across the gastrointestinal tract, but this possibility was not specifically addressed in the medical record. With the exception of *Nocardia* in 1 dog and *Clostridium perfringens* (found in conjunction with *E. coli*) in a second dog, the bacteria isolated in these dogs (*Pasteurella* spp. and *E. coli*) were typical of infecting organisms found in other causes of pyothorax. Therefore, the precise mechanism for development of pyothorax in these dogs remains unclear.

Contrary to our secondary hypothesis, septic pleural fluid was found in both cats and dogs despite the common use of antimicrobials befores sampling of fluid for culture, and positive bacterial culture results were obtained in 11/29 cats and 20/60 dogs that had received antimicrobials. This is an important observation clinically because empirical antibiotic treatment is commonly administered when pyothorax is suspected. It was not possible to determine the dose, duration, and timing of antibiotic treatment before thoracocentesis. Some animals in our study did receive effective doses of antimicrobials, given that 6/44 dogs and 2/27 cats had negative growth of bacteria despite having cytology results compatible with sepsis. The viability of intracellular bacteria cannot be assessed cytologically and, theoretically, visualized bacteria might have been killed by previous antimicrobial administration. New methodology for identification of bacteria by next generation sequencing could become an important modality for documenting infecting species in these cases, as well as in cases with idiopathic pleural effusion.^32^

Some studies have reported monomicrobial infection in the majority of both cat^4^ and dog^3^ cases of pyothorax; however, the majority of cases here and in other studies^2^, ^10^, ^18^ had polymicrobial infection, which emphasizes the need to perform both aerobic and anaerobic cultures. Antimicrobial susceptibility testing is essential to ensure efficacy of treatment against all infecting organisms. In our study, gram‐positive bacteria and *Pseudomonas* species were commonly resistant to enrofloxacin in contrast to a previous study^18^ that reported 85% susceptibility to enrofloxacin. In our cases, 67% of streptococcal and *Pseudomonas* spp. were susceptible to marbofloxacin, whereas another study found resistance to marbofloxacin in 71% of cases.^4^ With the exception of *Staphylococcus* spp., most organisms isolated in our study retained susceptibility to a potentiated penicillin drug (e.g., amoxicillin/clavulanate), which refuted our hypothesis that previous antimicrobial treatment would result in increased bacterial resistance. Interestingly, aerobic bacterial isolates found in our study were commonly susceptible to bacteriostatic antimicrobials (chloramphenicol and doxycycline), which also have efficacy against some anaerobic bacteria. Whether these drugs should be used instead of the recommended treatment with a penicillin derivative and fluoroquinolone^37^ to avoid development of antimicrobial resistance, requires further study.

Limitations of our study include those common to retrospective studies, which lack a prescribed method of data collection and assessment, particularly because the criteria for inclusion in our study were based on clinical diagnoses. Although our clinical pathology service has specific cut‐off values for protein concentration and cell counts in exudates,^13^ some animals met only 1 of those criteria. Many cases were excluded from analysis because pleural fluid was not submitted contemporaneously for both culture and cytology, and it is likely that our study was underpowered to determine significant differences between variables for specific etiologies. Although some animals fit well into categories of bite wounds or foreign bodies based on defined criteria, a number of animals had less definitive findings, which resulted in the category of suspected penetrating injury (inside‐out or outside‐in). These issues could be more appropriately addressed in a prospective study. We cannot exclude the possibility that pleuropneumonia developed in some animals with foreign bodies or bite wounds as a cause for pyothorax, although these are not reported as risk factors for aspiration.^38^, ^39^ The occurrence in some dogs of pyothorax in conjunction with systemic disease remains intriguing. It seems unlikely that immunosuppressive drug treatment led to empyema or predisposed dogs to this condition. Multiorgan dysfunction and translocation of bacteria might have played a role in some dogs, but this possibility could not be discerned in a retrospective study. Finally, cats and dogs were assigned a cause for pyothorax based on evidence in the medical record, which is subject to interpretation. Theoretically, a cat with pyothorax could be assigned an etiology of bite wound based on multi‐pet household and history of fighting only. Although not the case here, the uncertainties of defining an etiology based on retrospective data must be considered when interpreting our results.

In summary, in our study, pyothorax in dogs was most commonly associated with foreign body inhalation whereas pyothorax in cats was related to bite wounds or traumatic injury to the thorax. Interestingly, we found pyothorax in conjunction with various systemic diseases in some dogs, which represents a unique finding. In many studies, the etiology of pyothorax remains obscure in cats and in dogs.^10^, ^11^, ^18^ However, comprehensive medical record review here yielded confident assessment of the probable cause of pyothorax in all but 3/89 cases, even though almost 25% of all cases were narrowed down to penetrating injury alone. The inability to assign a precise cause represents a limitation of our study, but represents an opportunity for future prospective studies of pyothorax. Improved scrutiny for upper respiratory tract signs and confirmation of lower airway bacterial species that match pleural organisms would provide more confidence in a role for parapneumonic spread as a cause for pyothorax in cats.

## CONFLICT OF INTEREST DECLARATION

Authors declare no conflict of interest.

## OFF‐LABEL ANTIMICROBIAL DECLARATION

Authors declare no off‐label use of antimicrobials.

## INSTITUTIONAL ANIMAL CARE AND USE COMMITTEE (IACUC) OR OTHER APPROVAL DECLARATION

Authors declare no IACUC or other approval was needed.

## HUMAN ETHICS APPROVAL DECLARATION

Authors declare human ethics approval was not needed for this study.
